# Supervising students in a complex nursing practice- a focus group study in Norway

**DOI:** 10.1186/s12912-021-00693-1

**Published:** 2021-09-15

**Authors:** Ann-Chatrin Leonardsen, Siri Brynhildsen, Mette Tindvik Hansen, Vigdis Abrahamsen Grøndahl

**Affiliations:** 1 Østfold University College, Postal box code 700, 1757 Halden, Norway; 2 Østfold Hospital Trust, Postal box code 700, 1757 Halden, Norway

**Keywords:** Registered nurse, Intellectual disability nurse, Supervision, Clinical practice, Focus groups

## Abstract

**Background:**

The supervisory role of registered nurses and intellectual disability nurses will be even more essential in the future, to support the education of competent newly graduated candidates. To our knowledge few studies have explored nursing student supervisors’ perspectives on supervision across primary- and hospital healthcare services and also across nurse educational programs. The aim of the current study was to investigate supervisors’ perspectives on supervising from different clinical settings, and across registered nurses’ and intellectual disability nurses’ clinical practice.

**Methods:**

The study had an exploratory and descriptive design. The study was conducted within one university college catchment area in Southeastern-Norway. Eight focous group interviews were conducted in primary healthcare (n = 4) and hospital (n = 4) wards. A total of 31 registered nurses and three intellectual disability nurses participated. Hsieh and Shannon’s conventional content analysis was used to analyze the data.

**Results:**

Participants across primary- and hospital healthcare agreed that clinical practice was complex, and required that students gained competence in both technical and non-technical skills. Moreover, needed skills were described both as general and arena specific, and as both basic and advanced. Participants perceived that technical and non-technical skills together, ideally should lead to students being able to «see the person» behind the patient.

**Conclusions:**

Supervisors emphasized the challenges of supervising students in a complex nursing practice. Students should gain both procedural competence and an ability to provide person-centred care, and this challenged the supervisors’ own competence. Our findings indicate a need to support supervisors, to enable them to meet these challenges.

## Introduction

The World Health Organization (WHO) states that nurses will be key to the achievement of universal health coverage [1]. Nurses play a critical role in health promotion, disease prevention and healthcare delivery both in the community and in hospitals. There is a global shortage of health workers, and the WHO estimates that the world will need an additional 9 million nurses and midwives by the year 2030 [1]. In the U.S., Registered Nursing (RN) is listed among the top occupations in terms of job growth through 2029 [2, 3]. This is also the situation in Norway [4].

Professional capability in nursing is essential for quality of care, including patient safety and satisfaction [5]. Torabizadeh et al. [6] describe professional capability in nursing as a broad concept including a wide range of individual abilities and characteristics, and comprising a combination of knowledge, skills, personal qualities and understanding employed in an effective manner in both predictable and unexpected situations. In addition, the increasing implementation of technology enabled care in healthcare services leads to a need for reorganization of healthcare personnel’s working methods and services [7]. In addition, digital competence among healthcare personnel is still a challenge [8], and nurses express a need for more knowledge related to digitalization in healthcare services [9].

## Background

In Norway, nursing services are provided by RNs or Intellectual Disability Nurses (IDNs). The education that qualifies for accreditation as a RN or an IDN is a three year bachelor program (180 European Credit Transfer and Accumulation System, ECTS). In the RN program, 90 ECTS are devoted to clinical studies, of which between 32 and 42 weeks are spent in primary healthcare and in hospital [10]. The IDN cirruculum includes approximately 30 weeks of clinical studies. The IDN profession has changed over time [11], and to date no evidence-based interventions to guide IDN practice exist [12].

Clinical studies are a major component of both the RN and IDN education curriculum, and are considered a very important learning environment for the development of practical skills competence [13, 14]. Clinical studies provide nursing students with varied, but also limited opportunities to practice practical skills, due to a high degree of specialization and introduction of innovative medical technologies in healthcare services [15, 16]. Students express that the quality of their learning experiences are dependent on the behaviour and attitude of others, and the role of the clinical supervisor is influential in the overall experiences of students [17]. Still, supervisors in the clinical studies experience challenges with balancing the responsibility for both patients and the student, they have limited time to supervise, and they request closer collaboration with the educational institution [18, 19]. Healthcare services in general, and nursing services in special, are facing future challenges related to the increasing amount of elderly, persons with chronic diseases, reorganization of services due to limited hospital capacity and implementation of technology enabled care [15, 20, 21].

In this complexity, the supervisory role of RNs and IDNs will be even more essential to support the education of competent newly graduated candidates [22]. As RN and IDN educators (all female, one with a Ph.d. and one pofessor), with several years of clinical experiences, to our knowledge few studies have explored RN and IDN supervisors’ perspectives on supervision across primary- and hospital healthcare services and also across RN and IDN education programs.

## Methods

The purpose of this study was to investigate supervisors’ perspectives on supervising from different clinical settings, and across RN and IDN clinical practice.

### Design

The study had an exploratory and descriptive design, exploring how RNs and IDNs experience supervision, dependent on their backgrounds, interests, and interpretations [23]. Focus group interviews have the potential to discover the complexity of a study purpose, and allow for engagement between participants that can lead to more enriched descriptions [24], hence this was assumed an appropriate data collection method. The study adheres to the Consolidated Criteria for Reporting Qualitative Research (COREQ) [25].

### Setting and sample

The study was conducted within one university college catchment area, in a county in Southeastern-Norway with approximately 320.000 inhabitants. This area covers several primary healthcare wards in for example nursing homes, home-based nursing or homes for persons with intellectual/physical disability, as well as one hospital trust comprising both acute and elective hospital wards, where RN and IDN students complete their clinical studies. Yearly, about 160 RN students and from 45 to 95 IDN students graduate from the university college. Pragmatically, we aimed at including participants to four focus group interviews in primary healthcare wards, and four focus groups in hospital wards. Inclusion criteria were RNs and IDNs who had supervised students during the past two years. We aimed at a maximum variation sampling strategy [26], including a variation in types of wards, both in primary healthcare and in hospital. Through discussions in the research group, we agreed on inviting participants from (1) a nursing home, (2) a homed-based care ward, (3) a home for persons with intellectual/functional disabilities, and (4) services for persons with concurrent substance abuse and/or mental health disorders in primary healthcare, and from a medical/ surgical, an orthopedic, a pediatric and a ward for persons with concurrent substance abuse and/or mental health disorders in hospital. Selection of wards were based on input from the «Development Center for Nursing Homes and Home-based nursing» in the county. Initially, we contacted the managers in theselected wards, asking if they were interested in participating in our study. The managers then recruited RNs or IDNs who fulfilled the inclusion criteria, assumed to be information-rich, and who agreed to participate in a focus group interview.

### Datacollection

We developed a thematic interview guide based on earlier research [17, 18], as well as on iterative discussions among the researchers (see Table 1). The guide focused on themes such as students’ practical skills and how supervisors meant students best could learn these, supervisors’ expectations both related to students’ skills and to students’ preparedness, as well as on the supervisory role.

**Table 1:** Thematic interview guide

**Theme 1. Students’ practical skills** Could you please discuss your reflections regarding students’ practical skills? Which practical skills do you think students should learn while in clinical placement? Why? Which practical skills do you think students should learn in the university college? Why? Which procedures are needed in your ward? Could you please describe a situation where a student demonstrated the level of competence you expected? And the oposite?
**Theme 2. Expectations** Could you please discuss your expectations to students’ skills regarding practical procedures? Could you please describe a student that was well prepared before clinical placement What could have been improved regarding students’ preparedness?
**Theme 3. Supervisory role** Please discuss your experiences with supervising practical skills How do you approach a supervisory setting?

 The interviews were conducted nearby the participants’ workplace, but outside the actual ward, and preferably during the participants’ working hours. During the interviews, two researchers were present. One of the researchers fascilitated the interview discussions, while the other one wrote down initial impressions, and verbal and non-verbal expressions. Participants were familiar with the aim of the study, as well as with the background of the researchers. The interviews were conducted by a total of seven researchers (five RNs and two IDNs), including the authors. One researcher with extensive experience from focus group interviews participated in all interviews. The interviews lasted from 30 to 70 min (mean 50 min). The interviews were digitally recorded, and transcribed verbatim by an external transcriber, who had signed a non disclosure agreement.

### Analysis

We used Hsieh and Shannon’s conventional content analysis to analyze the data [27]. The analysis was conducted in four phases. In phase one, the analysis started with reading and re-reading of the transcripts, writing down and discussing initial ideas in a research group consisting of RN (n = 4) and IDN (n = 2) educators, as well as RNs from primary- (n = 3) and hospital (n = 3) healthcare services. In this phase, the notes from the interviews were included in discussions and development of initial ideas. The notes were also kept close during the iterative process of analysis from phase two to four. In phase two, the transcripts were inductively and thoroughly read, and key thoughts or concepts were highlighted (SB and VAG). The third phase included collating key thoughts or concepts into codes (ACL, SB, MTH and VAG). Finally, the codes were sorted into different categories depending on how they were related and linked (ACL, SB, MTH and VAG). The categories where then collated into an overarching theme. The development of categories/theme was also discussed among all authors in an iterative process moving back and forth the phases, untill consensus was reached. Table 2 gives an example of the analysis process.

**Table 2:** Example of the analysis process

Inductive highlighting of concepts, transcript	Codes	Categories	Overarching theme
I think about the basics, from scratch, think about young persons that may not ever touched another person before. From the first meeting, to basic care, how to approach another person, to the more advanced procedures…Basics including feeding, personal hygiene, care, to injections, blood sampling or handling central catheters	Basic skills Touching another person First meeting Basic care More advanced procedures From feeding to handling central catheters	Development of procedural competence	Students need to learn to see the person in the complexity of nursing practice

### Ethical consideration

 A willing, informed, written consent to participate was obtained from all participants. Due to the nature of a focus group study, participants were informed that they could not withdraw from the study in retrospect. All methods were carried out in accordance with relevant guidelines and regulations. In Norway, the Regional Committees for Medical and Health Research Ethics (REC) are responsible for approving medical and health related research projects. When patient data is not involved in the project, the study most commonly do not need approval from REC to perform the study. The study was assessed by REC, and judged not needing an ethical approval (reg.nr 2018/1281). The study was approved by the Norwegian Centre for Research Data (NSD, project no. 951,914), to ensure proper datacollection and storage.

## Results

Eight focus group (FG) interviews, including two to seven participants, were conducted in the periode August to December 2018. A total of 31 RNs and three IDNs participated in the interviews, only one was male. Table 3 gives an overview of participants’ characteristics.

**Table 3:** Characteristics of the study participants (*n* = 34) and setting for focus group interviews (*n* = 8)

Age in years, median (range)	40 (23–58)
Years of experience, median (range)	7.5 (1–35)
Supervision experience^a^	2–20
Formal education as supervisor, n=	16
Focus group 1	Nursing home
Focus group 2	Home-based nursing
Focus group 3	Home for persons with intellectual/functional disabilities
Focus group 4	Services for persons with concurrent substance abuse and/or mental health disorders
Focus group 5	Combined medical-surgical ward
Focus group 6	Orthopedic ward
Focus group 7	Pediatric ward
Focus group 8	Ward for persons with concurrent substance abuse and/or mental health disorders

Years of experience as a registered nurse or an intellectual disability nurse respectively. ^a^Supervision experience: reported as number of students supervised. Some reported to have supervised «many» students, hence median is not reported.

Through analysis, we identified one theme relating to the purpose of the study: «Students need to learn to see the person in the complexity of nursing practice», with subthemes «development of procedural competence» and «focus on person-centredness». Focus group (FG) 1–4 represent participants from primary healthcare services, while FG 5–8 represent hospital participants.

### Students need to learn to see the person in the complexity of nursing practice

Participants across primary- and hospital healthcare agreed that clinical practice was complex, and required that students gained competence in both technical and non-technical skills. Moreover, needed skills were described as both general and arena specific, and as both basic and complex. Participants perceived that technical and non-technical skills together ideally should lead to students being able to «see the person» behind the patient. All of the participants expressed a desire to fascilitate and optimalize students’ learning outcomes, even though their daily work did not always allow them to. We could not identify any differences between RNs and IDNs regarding these issues.

#### Development of procedural competence

Technical skills were interpreted in all focus groups as procedural competence, an ability to perform specific procedures. When supervising, the participants emphasized that students should learn both simple or basic procedures, as well as advanced procedures where the entirety was in focus. Simple or basic procedures were examplified as «bed making, tooth-brushing, or supporting personal hygiena», as well as «to measure heartrate, blood pressure, count respiratory rate or to assess NEWS (national early warning score)», both in primary healthcare services and in hospital. Drug knowledge and -administration were also mentioned by the participants in several focus groups as basic skills.

Participants working in the hospital emphasized the need for students to learn specific procedures such as injections, peripheral venous cannulation, blood- or urine sampling, while these procedures were not in focus in the primary healthcare groups. In focus group (FG) five, a participant also added documentation and technological competence as basic skills:

*«… general vital measurements, blood samples, urine samples, documentation, e-link… That kind of things, medication, adding doses…» (FG 5)*.

Participants also described arena specific procedures that they experienced the students would be exposed to during their clinical practice. The descriptions showed that students meet many different patient groups and a need for many different technical skills during their education. For example, participants from services for persons with concurrent substance abuse and/or mental health disorders emphasized the need for students to develop communication- and environmental working skills, as well as to implement their knowledge about legal rules and legislations in their practice. In FG 8, one of the participants stated:

«…*to have a basic understanding for new legislations, for example, why you do what you do…Because now, we don’t do anything without legal basis, and that’s new to us…»*.

In addition, participants emphasized that students needed to be able to adjust their procedural approach to the concrete patient. For example, in FG 7, participants described the difference between adults and children:

*«It is very different to work with children compared to adults…if you take a blood pressure, and you get the answer of the blood pressure, it is maybe something quite different you will see if you take the blood pressure in an adult» (FG 7)*.

The participants described different approaches to supervision dependent on the context, how far the student had reached in the educational program and also on whether the student had earlier experience with working in healthcare services. Even if they emphasized that students should take initiative, they all expressed the importance that they as supervisors invited the student in to different learning situations. Moreover, all focus groups described a progression throughout the practice periode, where the supervision changed from «showing the student» to the supervisor withdrawing from the situation. A participant in FG 6 prompted:

*«First watch, then try under surveillance, and then try alone»*

Participants also described supervision both before, during and after practical procedures. Before, they asked the student how they planned to perform the procedure to fascilitate reflection. They also emphasized the importance of role clarification before entering the patients’ room. Participants stated that it was important that they themselves had the needed procedural competence, and that patients should not experience an extra burden because of the student.

#### Focus on person-centredness

 In addition to procedural competence, non- technical skills like how to meet other people, to show respect and preserve the patient’s dignity, was by the participants in all groups described as very important. This was assumed as a basic, non-technical skill. As described in FG 5:

* «When I hear basic, I think that how you act and how you meet the patient and the relatives, that’s basic»*.

The participant continued:

*«to see the person, that you are able to see the whole person, how to meet them and that one can see what they can manage and what they can not»*.

This was supported by the other participants in the focus group, and also through discussions in all of the other groups. They all emphasized that the most important, also when performing procedures, was to be aware of the person exposed to the procedure. In FG 8, one participant stated:

*«Have to interpret that it’s the whole patient they should assess. The entirety, not just the ankle»*.

Most of the participants also acknowledged that this could be difficult for some students, especially early in the educational program. Then, participants were aware of their responsibility as supervisors. As stated in FG 2:

«*… when the student is unsecure about a procedure, the student is sometimes allowed to just focus on that, and I take the other role. That means, to take care of the patient, communication … And then the student is allowed to concentrate on the first element first, right»*.

This was supported in discussions in most other focus groups as well.

## Discussion

This study gives insight into supervisors’ perspectives on supervising RN and IDN students in both primary healthcare- and hospital wards. Across settings and educational background supervisors emphasized the complexity in nursing practice, and how supervisors needed to contextually adjust their approach to the student. Students’ clinical practice included a need to gain both procedural competence as well as an ability to provide person-centred care, which also challenged the supervisors’ competence.

Our findings show that students have their clinical practice in many different settings, requiring many different procedural skills. Supervisors described that students needed to learn both basic and advanced technical skills, as well as both general and arena specific skills when in clinical practice. A recent study found that RNs and IDNs perceive that students should learn both basic and advanced technical skills in the university college, and then practice further in clinical practice [28]. Moreover, another study showed that RNs in primary healthcare receive training in specific technical procedures such as handling of ventilators, tracheostomia, palliation or dialysis [9]. This complexity has also been indentified in international studies (e.g. [29–31]). These studies indicate an incongruency related to the perception of where students should learn technical skills: in school or in clinical practice, and also if they really need to «learn everything» when they are students.

Moreover, studies indicate that students have negative experiences concerning the behaviour and competencies of the supervisors [15, 29]. In addition, students experience incongruence amongst clinical supervisors regarding technical procedures [32, 33]. This complexity of nurses’ clinical practice is supported by Ravik et al. [13], who state that practical nursing skills are complex due to involving technical, theoretical and practical aspects, caring perspectives adjusted to both patient and circumstances, as well as ethical and moral considerations. Performing practical skills on patients is assumed to be more efficient to reach a more in-depth understanding than what students achieve through simulation or training in skills centres [34], hence it has been emphasized that policymakers should focus on improving the clinical environment, enabling for the professional development of students [35].

Participants perceived that technical and non-technical skills together ideally should lead to students being able to provide person-centred care. To supervisors, this challenged their own competence as well, requiring them to fascilitate good learning situations. Bos et al. [29] found that supervisors felt abandoned by their managers, colleagues and teachers from universities. Moreover, they experienced ambivalence due to simultaneously being supervisors for students and carrying out their daily work with patients. All of the participants in our study expressed a desire to fascilitate and optimalize students’ learning outcomes, even though their daily work did not always allow them to. Nevertheless, only half of them had formal education in supervision. Ewertsson et al. [36] emphasized a need for creating continuity between the ways that experiences are organized across the settings of learning (university-based and clinically based learning) to enhance nursing students’ learning and socialization into practical skills. A recent study from Denmarkimply that managers should choose supervisors on the basis of their motivation and their voluntary wish to partake the role [15]. Both the clinical practice and the supervision process is complex, requiring adjustment to procedure, patient and setting, as well as to the individual student.

Various types of knowledge must be mobilized due to the complexity of clinical practice, namely intrapersonal, interpersonal perceptual, moral/ethical, experimental, practical, scientific and contextual [37]. Hence, RN and IDN students have a lot to learn to be able to meet this complexity. The concept of care complexity has been widely used in the field of healthcare for many years, but is still not clearly defined [30]. Three factors have been closely related to care complexity: patient factors, nursing staff factors, and equipment or organizational factors [38]. Nursing factors have been described to include work experiences, education, knowledge and operational skills of caring and communication skills [38, 39]. This has also been described as «capabilities» in professional nursing: individualized-based capability, clinical judgment-based capability, research-based capability, inter-professional-based capability, clinical and practical-based capability, and ethical practice-based capability [5]. Hence, it is not only clinical practice that is complex, but also the nature of supervising.

In Norway, national guidelines for RN and IDN education were adopted in 2019 [40, 41]. Only the RN guidelines specifies that «the educational institution must offer education in supervision, and the parts (the educational institution and the clinical practice ward) must together develop a plan for conduction of such education». Our study support the need for such education for both RNs and IDNs.

### Methodological considerations

Within the qualitative research design lies the limited ability to generalize findings. Nevertheless, a strength of this study is that the focus group interviews were conducted in four different primary healthcare wards and four different hospital wards, respectively. Hence, this increases the transferability of findings to similar settings. Moreover, the study was conducted in a Norwegian setting, and one may argue that our findings are only valid in healthcare settings similar to those in Norway. Nevertheless, our findings are supported by international studies. In addition, including participants from different wards, and from both rural and central areas of the county may enhance the dependability of the findings.

Since participation was based on willingness to participate, and recruitment of participants were left to the managers in the selected wards, we were able to include three IDNs only. Even though, some of the RNs also supervised IDN students, and provided rich information about both RN and IDN students’ clinical practice. Of course, this may have an impact on the transferability of findings to an IDN setting.

A limitation of this study could be that the focus groups consisted of RNs/IDNs from the same workplace, resulting in that there were few controversies. Nevertheless, this was not only within groups, but also across groups, indicating a consensus on the statements. Another limitation could be that all participants related to one university college only. Including participants from other parts of the country could have provided other information and aspects to the discussion.

Rigour was ensured through a systematic approach throughout the study, as well as iterative discussions within the research group. Two of the researchers independently initially coded the data, but all authors reached consensus on the final analysis. The entire research group increased the rigour of the study as the members discussed interpretations and explored different positions.

In this study, transcripts and/or the analysis were not returned to participants for comments or corrections. The validity of our findings could have been improved through letting participants read the transcript and analysis of findings.

## Conclusions

Both RNs and IDNs emphasized the challenges of supervising students in a complex nursing practice. Both our findings and findings in international studies indicate that students should gain both procedural competence and an ability to provide person-centred care, and this challenge the supervisors’ own competence. Our findings indicate a need to support supervisors, to enable them to meet these challenges. In Norway, national guidelines state that educational insitutions must offer education in supervision- in RN education. This should be included in the guidelines for intellectual disability nurses as well, and also both nationally and internationally.

## Data Availability

The datasets used and/or analysed during the current study are available from the corresponding author on reasonable request.
